# Statistics Relating to a Scarlatina Epidemic*Read before the Buffalo Medical Club, Feb. 2, 1881.

**Published:** 1881-03

**Authors:** Joseph Fowler


BUFFALO

### MEDICAL AND SURGICAL JOURNAL.

VOL. XX.—MARCH, 1881.—No. 8.

### Original Communications.

### STATISTICS RELATING TO A SCARLATINA EPIDEMIC.*

BY JOSEPH FOWLER, M. D.

LATE in the year of 1873 scarlatina appeared in Buffalo, assumed an epidemic form, and continued till the close of 1875, being attended with an alarming mortality.

I have therefore prepared the following series of mortuary tables with the hope of ascertaining in what portion of the city this epidemic found its greatest number of victims; what localities were comparatively exempt, if any; whether unhealthy situations did actually increase the mortality, and the nature of the local influences, etc.

For this purpose I examined carefully the official mortuary records, and located the wards in which each case occurred in such a manner that the mortality percentage of any ward could be compared with that of any other, and have made a special mark upon a map of the city of Buffalo, indicating the location of each place as exactly as possible.

* Read before the Buffalo Medical Club, Feb. 2, 1881.

A census taken by the police force at a subsequent date afforded further facilities for determining with greater accuracy the percentage of deaths among the minor inhabitants of each ward in the city.

The epidemic in question began, as already stated, in October, 1873, two cases being reported for that month, and continued for a period of 27 months. This was an unusually long epidemic. Scarlatina had not appeared in this form for some years previous, and has not since been reported to any extent. This tends to corroborate an established law that small cities have more frequent visitations than large ones, while in the latter scarlatina and its allied exanthemata prevail almost without interruption, appearing in epidemic form at rarer intervals.

In the period alluded to, there were 714 deaths reported from scarlatina alone. I also observed, that during this time quite a large fatality among children from cerebral meningitis, convulsions, diphtheria and subsequently, "dropsy" and "debility," were reported as claiming many victims.

When an epidemic of this nature is raging, one can determine in his own mind, how many such cases were in all probability rendered fatal by the scarlatinal poison.

We do know, that scarlatina is often ushered in with convulsions, sometimes causing sudden death; that other fatal cases present symptoms of cerebral meningitis; that diphtheria is not infrequently the cause of death as a complication of scarlatina, and that "dropsy" and "debility" are common sequels of the disease.

I cite these facts preparatory to making the remark that whenever death is due to some complication of acute disease, the primary cause should be considered and so stated in the death certificate, although this is not always done. But, if allowance be made for such errors in diagnosis, it can be safely estimated that nearly a thousand children perished in this city during the period stated, from scarlatina alone.

But in the preparation of my tables, I can only calculate those cases where the cause of death is distinctly stated to be scarlatina. Of the entire number of deaths which I have noted, 372 were males, and 336 females, so far as sex could be ascertained. These figures compare favorably with other sets of statistics on the subject. Some mortality records would seem to show a predisposition of one sex to the disease, but others do not warrant any such conclusions.

Considering that in infancy male children predominate slightly, the figures here given would show that sex exercises no special predisposing influence. The following table gives the mortality during each month, from the beginning to the close of the epidemic:

MORTALITY OF EACH MONTH.

| | | | |
|---|---|---|---|
| 1873—October | 2 | 1874—December | 10 |
| " November | 14 | 1875—January | 51 |
| " December | 19 | " February | 39 |
| 1874 —January | 20 | " March | 29 |
| " February | 28 | " April | 27 |
| " March | 17 | " May | 10 |
| " April | 28 | " June | 2 |
| " May | 51 | " July | 11 |
| " June | 27 | " August | 5 |
| " July | 20 | " September | 6 |
| " August | 29 | " October | 5 |
| " September | 51 | " November | 5 |
| " October | 47 | " December | 3 |
| " November | 54 | | |

From this table it will be seen that the mortality reached almost its maximum in May, 1874, abating somewhat during the three succeeding months, and from that time was correspondingly high till April of the next year. From that month it gradually diminished, until it almost entirely ceased—December closing the period of calculation with three fatal cases. In the following table I have separated the ages into distinct periods, noting the sex and mortality of each:

| | Males. | Females. | Total. |
|---|---|---|---|
| Under 1 year........................ | 31 | 26 | 57 |
| From 1 to 2 years............... | 57 | 50 | 107 |
| " 2 " 3 " ............... | 63 | 45 | 108 |
| " 3 " 4 " ............... | 63 | 48 | 111 |
| " 4 " 5 " ............... | 33 | 41 | 74 |
| " 5 " 6 " ............... | 37 | 33 | 70 |
| " 6 " 7 " ............... | 23 | 15 | 38 |
| " 7 " 8 " ............... | 16 | 18 | 34 |
| " 8 " 9 " ............... | 11 | 10 | 21 |
| " 9 " 10 " ............... | 9 | 11 | 20 |
| " 10 " 11 " ............... | 5 | 12 | 17 |
| " 11 " 12 " ............... | 5 | 2 | 7 |
| " 12 " 13 " ............... | 2 | 6 | 8 |
| " 13 " 14 " ............... | 1 | 1 | 2 |
| " 14 " 15 " ............... | 0 | 4 | 4 |
| " 15 " 16 " ............... | 3 | 4 | 7 |

The law of an equally divided individual predisposition does not apply to scarlatina as to some other contagious and infectious diseases. Age exercises a decided predisposition influence over the production of the disease, as will be observed by consulting nearly all mortuary statistics.

Scarlatina is, of course, to be regarded as a disease of infancy, although occasionally contracted by adults. But the figures as here given suggest an interesting point concerning the age at which children are most susceptible to it, and in this they warrant conclusions different from those of most other observers.

The following I read in Ziemssen: "Haller observed a scarlatinous patient five months old; Fleishmann saw none under six; Eulenburg none under eight; I, none under five months. Senfft saw only one patient under one year; Gaupp only two; Böning none. According to Bokai, infants at the breast are rarely affected. The youngest patient that Voit saw was two and a half months old."

These notices prove that the predisposition to scarlatina, during the first year, is very slight on the continent.

Ziemssen, after comparing numerous observations, says, "that the number of cases, which have occurred during the first year, are so few in number, that we may safely assume for the latter (under one year) a very limited predisposition," and in closing, says, "nevertheless, even the *youngest individual* may have a predisposition to scarlatina." From this we might draw the conclusion, that the susceptibility of infants in one locality is much less than in another. It may be that the mortality percentage is greatly increased with our infants. Should it have reached 15 per cent. (a liberal estimate) from our table, the number of infants under one year of age, who were affected with the disease, would have reached nearly 400 in the period of our calculation. This is in great contrast compared with the observations of the authorities named.

This table compared more favorably in this respect with Murchison's statistics of 148,829 deaths from scarlet fever in 1847–55, and '61, in England, Scotland and Wales.

I have made another classification, giving the number of deaths in each ward in the city, and the total population of minors in the same, omitting the fractions for convenience of calculation.

| Wards. | No. of Deaths in each. | Total population under 21 years of age in each Ward respectively. | One died out of every |
|---|---|---|---|
| 1 | 71 | 7,031 | 99 |
| 2 | 13 | 2,350 | 170 |
| 3 | 33 | 5,193 | 154 |
| 4 | 27 | 4,038 | 149 |
| 5 | 103 | 12,685 | 122 |
| 6 | 78 | 7,802 | 100 |
| 7 | 85 | 9,978 | 117 |
| 8 | 26 | 3,309 | 127 |
| 9 | 16 | 2,543 | 158 |
| 10 | 45 | 4,553 | 100 |
| 11 | 43 | 5,508 | 128 |
| 12 | 9 | 3,076 | 341 |
| 13 | 16 | 1,543 | 96 |

It will be noticed that the wards range thus in the order of their mortality, viz: 13th, 1st, 6th, 10th, 7th, 5th, 8th, 11th, 4th, 3d, 9th, 2d, 12th.

To make a broader comparison, I have made two geographical sections of the sections of the city, and in them, am able to locate a number of cases which I could not otherwise include with certainty in the table of wards, owing to improperly filled certificates of death, Main street being the dividing line. Upon the west side thereof, being all that territory lying west of Main street, there reside 18,989 individuals under 21 years of age, and on the east side, which comprises the remaining city territory, there are 50,620 persons under 21 years, which exceeds the west side by 31,631. And notwithstanding the superior sanitary advantages and better drainage system in operation on the west side; notwithstanding its elevated situation, bounded upon one side by the lake and river shores, and although most of its dwellings are widely separated, and almost without exception possessing healthy surroundings, in spite of all these facts, the victims of scarlatina are unfortunately too numerous in this locality, and are often found in houses of the most wealthy.

In the tabulated statement just given, the Tenth ward is an excellent example of this.

In contrasting the sanitary condition of the east side with that of the west, where the surface is less undulating, which is more densely populated, its dwellings built so compactly, that their continuity is almost unbroken, which contains the principal work shops, tenements and slaughter houses of a great city, under such circumstances we should expect to find a largely increased mortality due in a measure to those causes.

Upon the west side, however, there was one death from scarlatina during this epidemic out of every one hundred and forty-seven of its minor inhabitants, while on the east side the death-rate was one in one hundred and sixteen.

The most unhealthy portion of the city is said to be that part of it traversed by the Hamburgh canal. This is an extension of the Erie canal; its width and depth being about the same as that, and its length nearly one mile long. It flows through the southeastern part of the city and receives the sewage of several hundred acres of closely populated territory, the refuse of slaughter-houses and many manufacturing establishments.

This gives off volumes of offensive gases, which can be readily detected in the atmosphere and yet in its vicinity the death-rate was not so very much greater than elsewhere.

It does not come within the scope of such a short paper to discuss the reasons of this. Undoubtedly other factors in the problem, which tend to lessen the mortality in certain localities, should be taken into consideration. But the inferences drawn, are based only on the facts as here stated, and as such are suggestive of knowledge yet to be gained upon this important subject, of the cause of scarlatina.

